# Tyrosinase and laccase-producing *Bacillus aryabhattai* TFG5 and its role in the polymerization of phenols

**DOI:** 10.1186/s12866-021-02258-3

**Published:** 2021-06-22

**Authors:** Iniyakumar Muniraj, Syed Shameer, Sivakumar Uthandi

**Affiliations:** 1 Biocatalysts Laboratory, Department of Agricultural Microbiology, Tamil Nadu Agricultural University, Coimbatore, Tamil Nadu 641 003 India; 2 Present address: Department of Agricultural Microbiology, AMRITA School of Agricultural Sciences, Arasampalayam, Coimbatore, 642 109 India

**Keywords:** *Bacillus aryabhattai* TFG5, Tyrosinase, Laccase, ARDRA, Polymerization of phenols, FT-IR

## Abstract

**Background:**

Tyrosinases and laccases are oxidoreductase enzymes that are used widely in the food, feed, textile, and biofuel industries. The rapidly growing industrial demand for bacterial oxido-reductases has encouraged research on this enzyme worldwide. These enzymes also play a key role in the formation of humic substances (HS) that are involved in controlling the biogeochemical carbon cycle, providing nutrients and bio-stimulants for plant growth, and interacting with inorganic and organic pollutants besides increasing carbon sequestration and mitigating greenhouse gas emission in the environment. The present study aimed to screen and characterize extracellular tyrosinase and laccase-producing soil bacteria that could be utilized in the polymerization of phenols.

**Results:**

Twenty isolates from different soil samples collected from forest ecosystems were characterized through ARDRA using restriction digestion with AluI, HpaII, and HaeIII restriction enzymes. The results of Hierarchical Cluster Analysis (HCA) revealed a 60 % similarity coefficient among 13 out of 20 isolates, of which, the isolate TFG5 exhibited only 10 % similarity when compared to all the other isolates. The isolate TFG5 exhibited both tyrosinase (1.34 U.mL^− 1^) and laccase (2.01 U.mL^− 1^) activity and was identified as *Bacillus aryabhattai*. The increased polymerization activity was observed when *B*. *aryabhattai* TFG5 was treated with phenols. The monomers such as catechol, p-Hydroxy benzoic acid, ferulic acid, and salicylic acid were polymerized efficiently, as evidenced by their FT-IR spectra depicting increased functional groups compared to the standard mushroom tyrosinase.

**Conclusions:**

The polymerization ability of *B. aryabhattai* TFG5 could be applied to phenol-rich wastewater treatment for efficient precipitation of phenols. Furthermore, tyrosinases can be used for enhancing the synthesis of HS in soil.

**Supplementary Information:**

The online version contains supplementary material available at 10.1186/s12866-021-02258-3.

## Background

Tyrosinase exhibits monophenol monooxygenase (EC 1.14.18.1) and o-diphenol: oxygen-oxidoreductase (EC1.10.3.1) activities. Laccase (EC 1.10.3.2) exhibits the ρ-diphenol:oxygen-oxidoreductase activity. Both the enzymes are grouped under oxidoreductases, which are present in most plants, fungi, bacteria, archaea, lichens and mammals [1–3]. Tyrosinase catalyzes the oxidation of L-tyrosine into melanin via dopaquinone and L-3,4-dihydroxyphenylalanine (L-DOPA); L-DOPA is administered in the treatment of Parkinson’s disease [4]. Laccase oxidizes a variety of phenols, non-phenolic substrates, and aromatic compounds and is the most suitable enzyme for industrial applications, such as oxidation of xenobiotic compounds and in the pulp and paper industry [5, 6]. In terms of oxidation activity, the main difference between laccase and tyrosinase is their substrate specificity. While laccase is capable of oxidizing syringaldizine and other phenolics as well as non-phenolic substrates, tyrosinase can oxidize only L-tyrosine. In addition, tyrosinase exhibits ceresolase activity, while laccase does not.

The commercial production of tyrosinase and laccase is limited to fungal sources. *Agaricus bisporus* mushroom is used for commercial tyrosinase production [7], while the white-rot basidiomycetes are well-known for the commercial production of laccase. Only a few bacterial tyrosinases are reported, predominantly from the *Streptomyces* sp. [8–10]. Tyrosinases are also known to occur in *Rhizobium* sp. [11], *Vibrio tyrosinaticus*, *Thermomicrobium* [12], *Azospirillum lipoferum* [13], CotE of *Bacillus* spore [14, 15], *Bacillus megaterium* [15] and *Bacillus aryabhattai* [16]. The presence of tyrosinase and laccase in soil microflora signifies the role of these enzymes in organic matter decomposition, and eventually, the formation of HS. The microbial oxidoreductases such as tyrosinases and laccases are reported to depolymerize the lignin present in the organic matter. Furthermore, the cross-linking of amino acids in the depolymerized lignin and the enhanced formation of the backbone structure of humic polymer in the decomposing soil are catalyzed by microbial tyrosinases [17–19]. In this manner, the microbial oxidoreductases contribute to soil carbon sequestration.

Moreover, in industrial applications, bacterial oxidoreductases are preferred over fungal ones as the former presents the advantages of faster multiplication rate, the convenience of commercialization, genome modifications, etc. Therefore, recent studies have focused on the screening and application of bacterial tyrosinases with significant secretory potential for industrial/soil applications. The present study involved the screening and characterization of laccase and tyrosinase-producing bacterial isolates from forest soils for use in the polymerization of the phenols in the soil or water environment. It was envisaged that the bacteria with dual enzyme secretion capability would be beneficial in the polymerization of phenolic molecules in wastewater. More importantly, research on such bacteria can unravel the mechanistic role of enzyme-mediated synthesis of HS in the soil so that the synthesis rate of humic substances could be increased, thereby increasing soil carbon sequestration. Previously, we attempted to understand the mechanism of formation and enhancement of HS from coir pith wastes using a tyrosinase produced by *B. aryabhattai* TFG5 [20]. The present study confirmed the identity of the potential soil bacteria that possess laccase and tyrosinase activity besides characterizing their role in the polymerization of phenols.

## Results

### Screening for tyrosinase and laccase-positive isolates

**Fig. 1:**
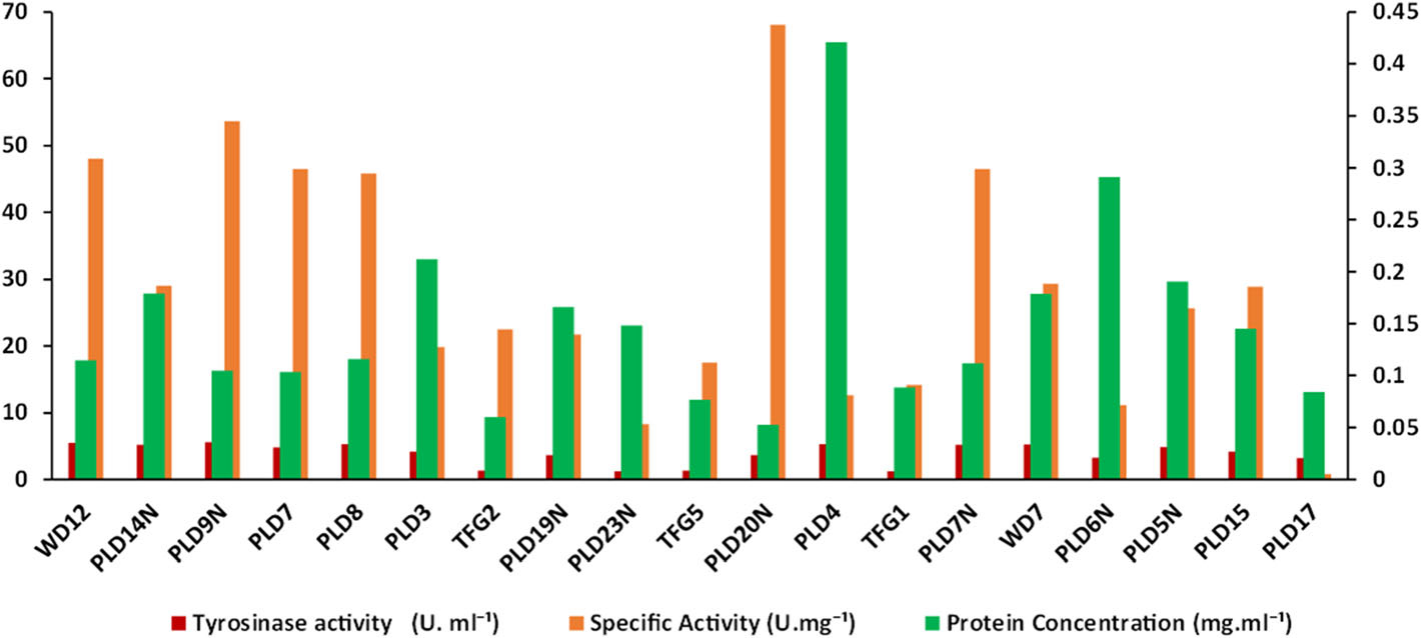
Screening of the isolates for tyrosinase production

The preliminary screening of isolates for tyrosinase and laccase activity revealed that among the 20 isolates screened, the maximum tyrosinase activity was exhibited by PLD9N (5.61 U.mL^− 1^), followed by WD12 (5.5 U.mL^− 1^) and WD 7 (5.23 U.mL^− 1^). The lowest activity of 1.23 U.mL^− 1^ was exhibited by isolate PLD23N. The maximum specific activity (68.04 U.mg^− 1^ of protein) was obtained for PLD20N, followed by PLD7N, while the lowest specific activity (1.74 U.mg^− 1^ of protein) was observed for PLD17 (Fig. 1 and Figure S1).

**Fig. 2:**
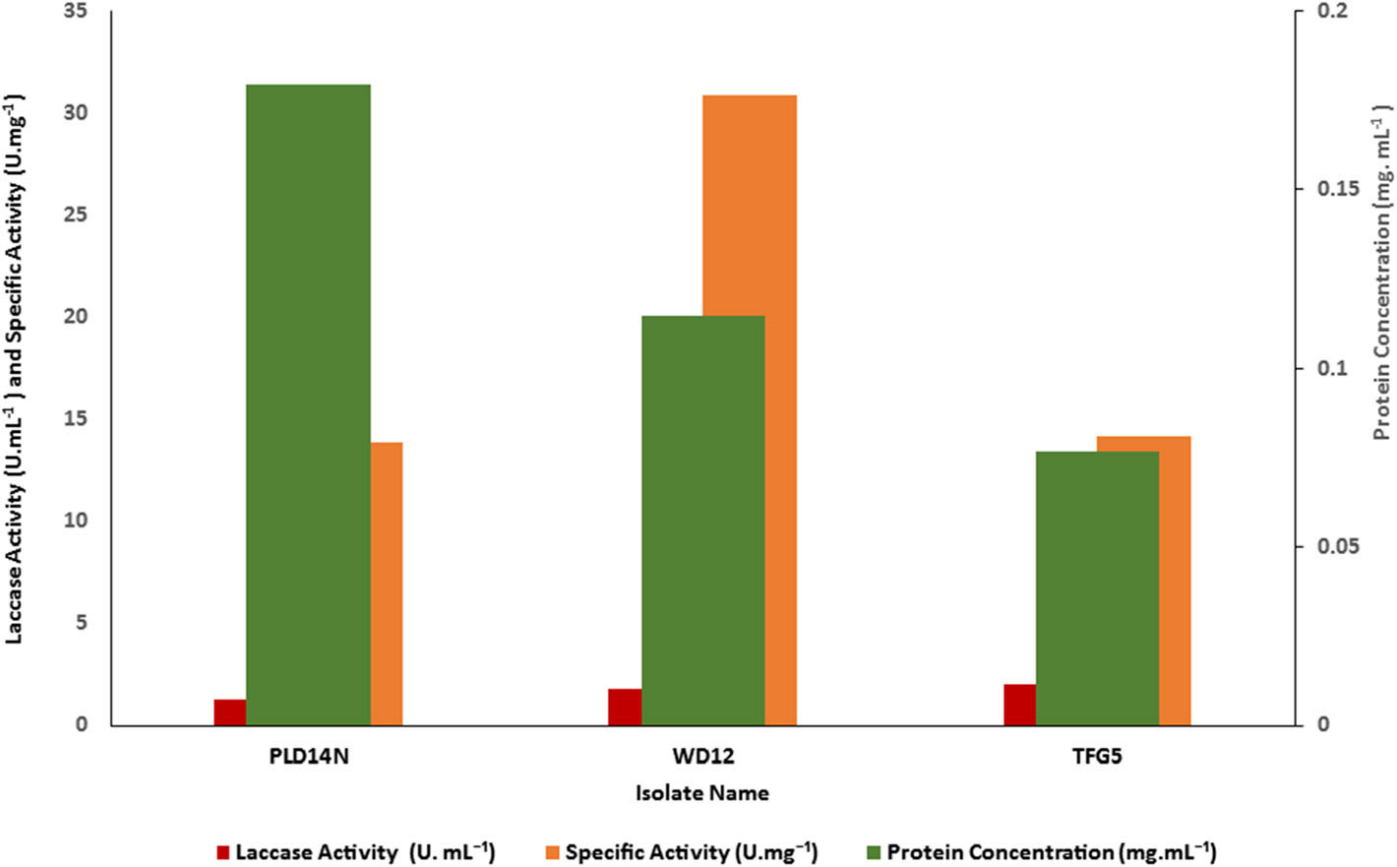
Screening of the isolates for laccase production

Among 20 isolates screened, PLD14N, WD12, and TFG5 produced both laccase and tyrosinase when cultivated in Crawford and L-tyrosine media, respectively, as explained in the materials and methods section. Among these three, the isolate TFG5 exhibited the maximum laccase activity of 2.01 U.mL^− 1^ and maximum specific activity of 14.15 U.mg^− 1^ of protein. The isolate WD12 exhibited a specific activity of 30.86 U.mg^− 1^ of protein, while its laccase activity was 1.79 U.mL^− 1^ (Fig. 2).

### Characterization of the isolates using ARDRA

Amplified Ribosomal DNA Restriction Analysis (ARDRA) is an efficient molecular tool for the identification of bacterial communities isolated from different locations. In the present study, ARDRA was employed to characterize all the twenty isolates. Genomic DNA was isolated from the isolates, followed by the amplification of 16 S rDNA (1500 bp) gene. The amplicon was digested with restriction enzymes AluI, HpaII, and HaeIII. The patterns of restriction for *Alu*I resulted in a clear and robust banding pattern between 100 and 600 bp (Figure S2). A strong banding pattern between 200 and 400 bp was observed for 14 among the 20 isolates, viz., lane 1–9, 12, 13, 15, 17, and 20. Such a banding pattern indicates that these 14 isolates belonged to a common group. Lanes 10, 11, 14, 15, and 19 presented distinct banding patterns between 100 and 800 bp, which suggested that these isolates belonged to a common group different from the one containing the other 14 isolates. A unique banding pattern between 100 and 600 bp was observed in lane 11 (Figure S2).

The overall restriction digestion patterns of *Hpa*II produced a strong and identical band between 100 and 600 bp. Three different and predominant banding patterns were observed upon digestion with *Hpa*II. Lanes 2, 3, 4, 5, 6, and 17 presented a banding pattern between 100 and 400 bp, demonstrating a similar digestion pattern. Similarly, lanes 1, 7, 8, 9, 13, 15, and 20, which presented a banding pattern between 100 and 400 bp, demonstrated identical banding patterns. The banding patterns different from the above two were observed in lanes 12, 14, 18, and 19. Among the all, lane 11 was more distinct from the others, with a banding pattern between 100 and 700 bp (Figure S3).

The predominant group in the case of restriction with *Hae*III included lanes 7, 8, 9, 15, and 20, with a banding pattern between 100 and 400 bp. Next to this, a banding pattern between 100 and 500 bp was present in lanes 2, 3, and 4. A 100–700 bp restriction band was observed in lanes 11, 14, and 19 (Figure S4).

**Fig. 3:**
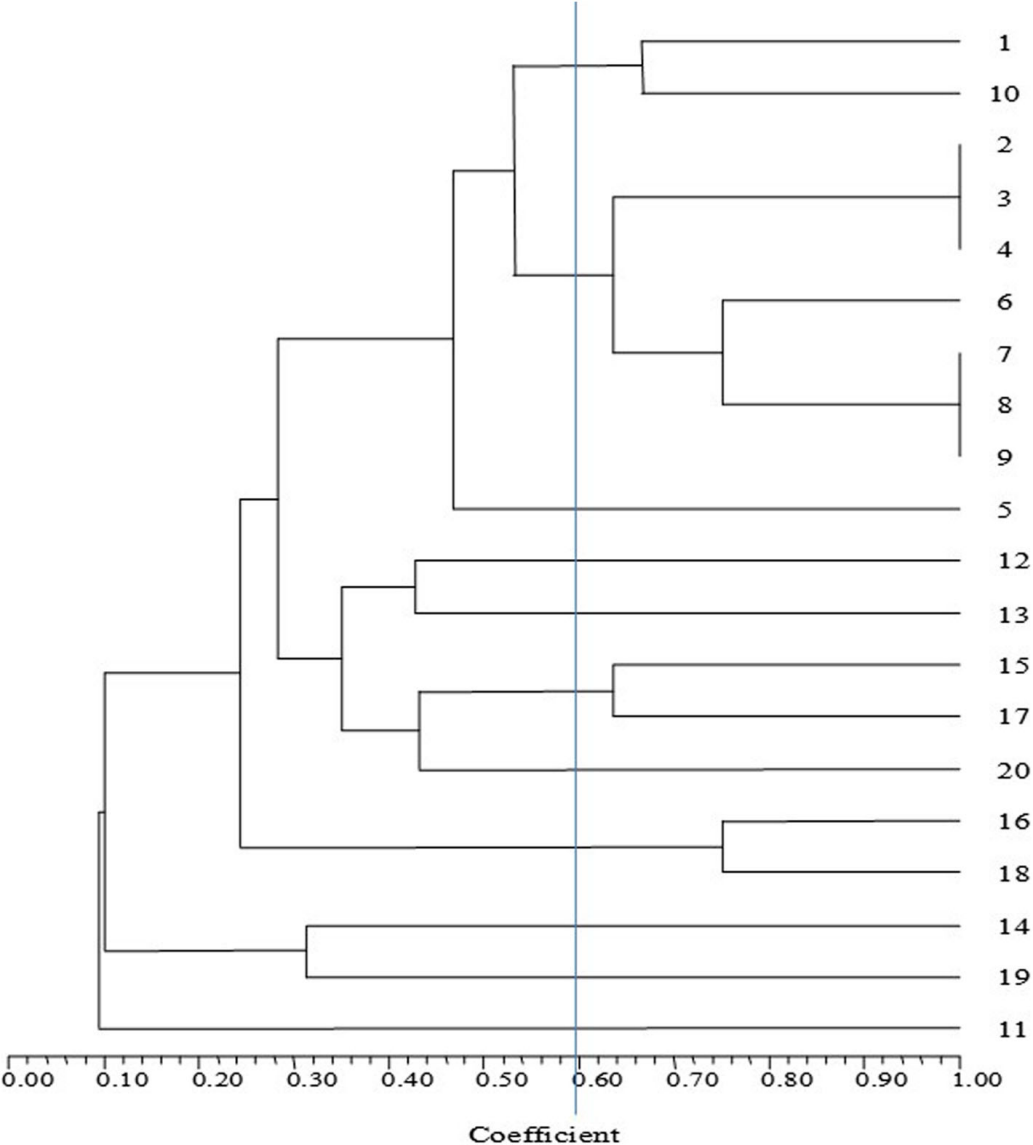
HCA dendrogram constructed using the ARDRA pattern of tyrosinase/laccase-positive isolates

**Fig. 4:**
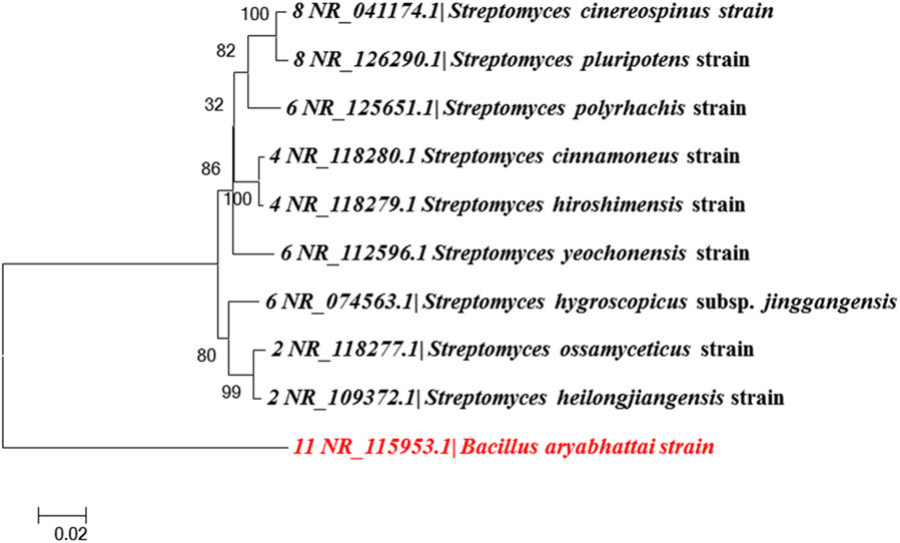
The phylogenetic tree constructed using the ARDRA pattern of tyrosinase/laccase-positive isolates

ARDRA profiling of three restriction enzymes revealed restriction digestion by *Hpa*II and *Hae*III was relatively similar to the tested tyrosinase/laccase-positive isolates. The grouping and the number of dominant bands were similar for both the enzymes (Figure S3 and S4). On the other hand, *Alu*I produced an entirely different banding pattern (Figure S2). Based on the restriction patterns, a dendrogram was constructed, and the phylogeny of the isolates was identified (Fig. 3). The similarity coefficient of 60 % was obtained for lanes 1, 10, 2, 3, 4, 6, 7, 8, 9, 15, 16, 17, and 18. Both lane 12 and lane 13 presented a similarity coefficient of 40 % and were identical isolates. Similarly, lanes 14 and 19 presented a similarity coefficient of 30 % and were identical isolates. Lane 11 was only 10 % similar to the other lanes, suggesting that this isolate was different from all the other isolates. The phylogenetic analysis of the tyrosinase/laccase-positive isolates revealed that the isolates having a similarity coefficient of over 60 % belonged to the *Streptomyces* sp. The distinct isolate of lane 11, with a similarity coefficient of 10 %, was identified as *Bacillus aryabhattai* possessing both tyrosinase and laccase activity (Fig. 4).

According to ARDRA profiling and phylogenetic relationship, a distinctively related bacterial isolate with a similarity coefficient of < 10 % was identified as *Bacillus aryabhattai* TFG5 based on the 16 S rRNA gene sequencing. The 16 S rRNA sequence of the isolate has been submitted to NCBI gene bank (GenBank accession number: KT956906). The literature on the bacteria, particularly *Bacillus* sp., with dual enzyme (tyrosinase and laccase) secretion capability, is scarce. Therefore, *Bacillus aryabhattai* TFG5 was selected for further analyses and characterization.

### Polymerization of phenols using *B. aryabhattai* TFG5

Table 1 describes the presence of different functional groups in monomers used along with their corresponding wavenumbers as observed in the FTIR spectra. The presence of a greater number of functional groups were observed in the extracellular protein of TFG5-treated phenols compared to the control ones (Table 1 and Figure S5). All the monomers used had functional groups representing alcohol at wave number 3328 cm^− 1^, alkenes at 1637.3 cm^− 1^, and amine salts at 2331 cm^− 1^ (Table 1); these functional groups represent polymerization. The transformation of aldehydes into alkenes and amine salts was observed in catechol and benzoic acid. In contrast, no such change was observed in ferulic acid and salicylic acid, suggesting that the extracellular protein from TFG5 exerted a transforming effect on the concerned polymers. The reverse was true for salicylic acid, in which the alkenes C = C present at the wavenumber 1637.3 cm^− 1^ were transformed into aldehydes at wave number 1737.55 cm^− 1^ due to the action of the extracellular protein from TFG5. In ferulic acid, no such transformation of alkenes was observed.

**Table 1:** FT-IR absorbance spectra and the corresponding functional groups of the monomers observed during polymerization

Monomers (3 mM)	With standard mushroom tyrosinase	Extracellular protein of *B. aryabhattai* TFG5	Control (without enzyme)
Catechol	578.5 cm^–1^: Halogens C-Br Strong-Stretching	584.325 cm^–1^: Halogen C-Br-strong-stretching	1368.25 cm^–1^: Alcohol OH Medium-Deformation
	1631.5 cm^-1^: Alkenes C =C Variable-Stretching non conjugated C =C	1637.27 cm^–1^: Alkenes C =C-Variable-Stretching Non conjugated C =C	1739.48 cm^–1^: Aldehydes C =O Strong-Stretching saturated aliphatic aldehyde
	3327.6 cm^–1^ : Alcohols OH Variable-Stretching hydrogen-bonded	2156.99 cm^–1^: Amine salts NH-Medium-Stretching-Secondary amine salt	
		3331.43 cm^–1^: Alcohol OH-Variable-Stretching hydrogen-bonded	
p-Hydroxy benzoic acid	570.8 cm^–1^Halogens C-Br Strong-Stretching	429.084 cm^–1^: Sulfur compounds S-S Variable-Stretching	1368.25 cm^–1^: Alcohol OH Medium-Deformation
	1637.3 cm^–1^: Alkenes C =C Variable-Stretching non conjugated C =C	1644.02 cm^–1^: Alkenes NH Variable-Stretching Secondary amine salts	1739.48 cm^–1^: Aldehydes C =O Strong-Stretching saturated aliphatic aldehyde
	3340.1 cm^–1^ Alcohols OH Variable-Stretching hydrogen-bonded	2162.78 cm^–1^: Amine salts NH Medium-Stretching Secondary amine salts	
		2355.62 cm^–1^: Amine salts NH Medium-Stretching Tertiary amine salts	
		3402.78 cm^–1^: Amines NH Weak-Secondary amine	
Ferulic acid	1637.3 cm^–1^: Alkenes C =C Variable-Stretching non conjugated C =C	556.363 cm^–1^: Halogens C-Br Strong-Stretching	1637.3 cm^–1^: Alkenes C =C Variable-Stretching Non conjugated C =C
	3340.1 cm^–1^ : Alcohols OH Variable-Stretching hydrogen-bonded	1325.8 cm^–1^: Alcohol OH Strong-Deformation	2112.6 cm^–1^: Alkynes CΞC Variable-Stretching Mono substituted
		1636.3 cm^–1^: Alkenes C =C Variable-Stretching Non conjugated C =C	3328.53 cm^–1^: Alcohol OH Variable-Stretching hydrogen-bonded
		2156.99 cm^–1^: Amine salts NH Medium- Stretching Secondary Amine salts	
		2338.2 cm^–1^: Amine salts NH Medium-Stretching Tertiary amine salts	
		2369.12 cm^–1^: Amine salts NH Medium-Stretching Tertiary amine salts	
		3331.43 cm^–1^: Alcohol OH Variable-Stretching Hydrogen Bonded	
Salicylic acid	1637.3 cm^–1^: Alkenes C =C Variable-Stretching non conjugated C =C	1214.93 cm^–1^: Alcohol C-O Strong- Stretching	1637.3 cm^–1^: Alkenes C =C Variable-Stretching Non conjugated C =C
	3340.1 cm^–1^ : Alcohols OH Variable-Stretching hydrogen-bonded	1367.28 cm^–1^: Alcohol OH Medium- Deformation	2112.6 cm^–1^: Alkynes CΞC Variable-Stretching Mono 3328.53substituted
		1737.55 cm^–1^: Aldehyde C =O Strong- Stretching saturated aliphatic aldehyde	3328.53 cm^–1^: Alcohol OH Variable-Stretching hydrogen-bonded
		2331.52 cm^–1^: Amine salts NH Medium to strong-stretching tertiary amine salts	
		3454.8 cm^–1^: Amines NH Weak- Stretching Secondary amines	
		3627.4 cm^–1^: Silicon compounds OH Medium-Stretching Broad peak	

## Discussion

Tyrosinases and laccases are enzymes occurring naturally in plants, fungi, bacteria, and mammals, with potential applications in various fields such as medical, pharmaceutical, industrial, and wastewater treatment industries [21]. Tyrosinases from *Agaricus bisporus* are available commercially and are used widely for different applications. Tyrosinases from bacteria are being explored currently, most of them belonging to the *Streptomyces* sp. [13]. Laccases are another group of important industrial enzymes, with the bacterial laccases preferred over the fungal ones in the industry due to several advantages of the former, including convenient genetic modification to improve their function, better performance in biosensor applications, etc. [22]. The identification of novel bacterial strains producing tyrosinase and laccase is necessary to meet the growing demand for these enzymes in various industries [8]. Therefore, in the present study, ARDRA and tetra-cutter restriction endonucleases were used to screen and characterize isolates of soil microorganisms with both laccase and tyrosinase activity. ARDRA is a useful tool for the identification of microorganisms based on the restriction digestion patterns of the PCR-amplified products, because of its speed compared to phenotypic identification, reliability, practical applicability, flexibility, and the possibility of identifying most bacteria. It is a simple and rapid tool that does not require costly equipment and depends on the type of restriction enzymes in rapid follow-up for environmental microorganisms [23]. ARDRA has been successfully applied to characterize bacterial communities of the activated sludge in domestic and industrial wastewater [23]. The identification of the *Lactobacillus* bacterial community in poultry was successfully achieved in a previous study using ARDRA [24]. In the present study, ARDRA of soil bacterial communities revealed a novel bacterial strain *B. aryabhattai* TFG5 that possessed both tyrosinase and laccase activity. The yields of tyrosinase in this bacterial isolate were much higher than those reported for other *Bacillus* sp., such as *Bacillus megatherium* strain M36 with a tyrosinase activity of 0.522 IU [25].

The extracellular enzyme secreted by *B. aryabhattai* TFG5 was used for the polymerization of phenols. According to the results presented in Figure S5 and Table 1, new functional groups were observed due to the polymerization of catechol and benzoic acid. Aldehydes were polymerized into alkenes and amine salts. Such a transformation of phenols into additional functional groups confirms the superior efficiency of this bacterial tyrosinase over the standard tyrosinase (Figure S5 and Table 1). A similar kind of polymerization was observed in the study reported previously, in which phenol was treated with fungal laccase from *Ustilago maydis;* and FT- IR analysis of functional groups, revealed that new functional groups were formed upon treatment of flavonoids with laccase and tyrosinase [26]. The polymerization of phenolic molecules is vital for the removal of excess phenolic contaminants in wastewater [21], while the polymerization of degraded lignin with amino acids enhances the decomposition process and the formation of soil humic substances [17, 27]. In an earlier study, tyrosinase-mediated humification of coirpith wastes was successfully achieved by extracellular protein from *B. aryabhattai* TFG-5 [20]. The extracellular enzyme was characterized using homology modeling and confirmed as a tyrosinase. Besides transforming standard phenols, the extracellular enzyme also transformed free phenols in coirpith wash water. Coirpith wastes when treated with the enzyme synthesized humic substances in a short time [20]. The polymerization of phenols also assists in dye removal and the antioxidant activity of human cells [28]. Similarly, the reverse transformation of aldehydes into alkenes signifies that the aldehydes formed due to the polymerization of organic matter in the soil are transformed into alkenes by the extracellular enzyme from TFG5. Therefore, it can be envisaged from the results that the extracellular protein of TFG5 is superior to the standard tyrosinase and capable of transforming the degradative products of organic matter.

## Conclusions

Therefore, the novel bacterial isolate *B. aryabhattai* TFG5 is a potential candidate that could be used in the polymerization of the phenols in wastewater and soil environments. The dual enzyme-positive nature of this bacterial strain may be utilized in the industries for the removal of phenol content and acceleration of humification in soils.

## Methods

### Screening of tyrosinase and laccase-positive isolates

Soil samples were collected from different locations, including termite fungal garden and decayed woods of the Forest College and Research Institute, Mettupalayam (Jakanari Forest Range) having Peelamedu soil series (latitude and longitude of 11.2891^0^ N,76.9410^o^E). The collected samples were stored at 4 °C until use. The tyrosinase- and laccase-producing bacteria were isolated from the soil samples using the modified protocol of Dalford. Briefly, the soil samples were serially diluted up to 10^-^^6^, followed by plating on the medium, which contained (g.L^− 1^): casein broth hydrolysate (10), K_2_HPO_4_ (0.5), MgSO_4_ (0.25), and L-tyrosine (1) for tyrosinase-producing bacteria (Dalford et al. 2006). The laccase-positive colonies were screened by streaking the pure colonies on the soil extract agar medium containing (g.L^− 1^) glucose- (1.0), K_2_HPO_4_ (0.5), soil extract 100 mL, and agar (15.0) with a pH of 7.0. After culture growth, the colonies turning dark brown on the L-tyrosine plate (Fig. 1) were tyrosinase-positive. The laccase-positive colonies were identified based on the rapid appearance of pink coloration over the colony upon adding a drop of 0.1 M Syringaldazine (SGZ).

### Tyrosinase and laccase production under submerged fermentation

All the twenty isolates were pre-cultured in a nutrient broth containing (w/v) glucose (0.8 %), meat extract (1 %), peptone from casein (1 %), tryptone (1 %), and NaCl (0.05 %) at 37 °C inside an incubated shaker at 200 rpm. After 18 h of incubation, the culture was transferred to the production medium containing (g.L^− 1^) casein broth hydrolysate (10), K_2_HPO_4_ (0.5), MgSO_4_ (0.25), and L-tyrosine, followed by incubation at 37 °C under 200 rpm shaking conditions [25]. Laccase production was monitored for all the isolates cultured in Crawford’s broth supplemented with 0.01 % CuSO_4_ and incubated as described above [5].

### Enzyme assay

All the cultures grown in respective growth medium induced with either L-tyrosine or CuSO_4_ were centrifuged at 11,200 ×g for 10 min in a refrigerated centrifuge (ThermoFisher, India) at 4 °C to obtain a clear supernatant without cells. The cell-free culture supernatant was used as the enzyme source. Tyrosinase activity in the supernatant was determined based on the oxidation of 1 mM L-DOPA (3,4-dihydroxy-L-phenylalanine) in 50 mM sodium phosphate buffer (pH 7.0) into dopachrome in the presence of 5 mM MBTH, which was quantified by the increase in the absorption at 475 nm (ε475 = 3.6 mM^− 1^ cm^− 1^) [29]. The laccase activity in the supernatant collected from Crawford’s broth was determined based on the oxidation of syringaldizine (0.1 M), which was quantified spectrophotometrically by measuring the change in the absorbance at 530 nm with a molar extinction coefficient (€ max = 3.6 × 10^4^ M^− 1^ cm^− 1^) [25, 30]. One unit (U) of enzyme activity was defined as the amount of enzyme required to oxidize 1 µmol of the substrate minute^− 1^.mL^− 1^ of the reaction mixture under standard assay conditions. The protein concentration of the enzyme sample was determined using Bradford’s reagent (Bio-Rad, California, USA) in accordance with the manufacturer’s instructions and with bovine serum albumin (BSA) as the standard [31].

### Genomic DNA extraction and amplification of 16SrDNA gene

In order to characterize the 20 isolates obtained from the forest soil using ARDRA, genomic DNA was extracted from the actively-growing cultures grown in LB broth, followed by total DNA extraction using the CTAB method. The DNA concentration was estimated, and the purity was determined using agarose gel electrophoresis. Appropriately diluted (20 ng.µL^− 1^) DNA was used for the amplification of the 16 S rDNA gene. The forward and reverse primers used were: 27f AGAGTTTGATCCTGGCTCAG and 1492r ACGGYTACCTTGTTACGACTT. The PCR reactions were performed in 20 µL PCR mixture, including 1X Taq buffer, 2.5 mM MgCl_2_, 0.5 µM of forward and reverse primers each, 0.25 mM dNTP mixture, and 3 U of Pfu DNA Polymerase (all from Fermentas, USA). The PCR conditions were: initial denaturation at 95 °C for 5 min; 35 cycles of denaturation at 95 °C, each for 1 min; annealing at 55 °C for 1 min; primer extension at 72 °C for 1 min followed by the final extension at 72 °C for 10 min. Amplification was performed in BioRad T100 PCR. The presence of the 1.5-kb PCR product size was visualized using 1.2 % agarose gel electrophoresis.

### Restriction digestion analysis

The amplified PCR products were characterized by digestion with three restriction endonucleases, namely, *Alu*I, *Hpa*II, and *Hae*III [32]. The restriction digestion was performed at 37 °C for 8 h in thin-wall tubes with a reaction volume of 20 µL containing 10 µL of the amplified PCR product, 10 U of restriction enzymes, and appropriate buffer. The restriction digestion reaction was stopped by the thermal inactivation of the enzymes at 65 °C. The restricted fragment patterns were run on 1.2 % polyacrylamide gels containing ethidium bromide solution (5 ŋg.µL^− 1^).

### Construction of dendrogram and phylogenetic tree

The number and position of the bands obtained from the 16 S rDNA restriction of tyrosinase/laccase-positive isolates were used for constructing a dendrogram. The similarity coefficient of 60 % was used as the threshold to discriminate the different species. The phylogenetic analysis and the construction of an unrooted tree were performed using the MEGA software version 3.0.

### Polymerization of phenols by tyrosinase

Solutions of monomeric phenols (2 mM) were prepared in 100 mM sodium phosphate buffer at pH 6.5 by following the prescribed method [33]. The monomers used were catechol, p-hydroxybenzoic acid, ferulic acid, and salicylic acid. Separately, a standard tyrosinase (10 U.mL^− 1^) from mushroom and an extracellular protein from *B. aryabhattai* TFG5 were compared. A negative control without the tyrosinase enzyme was also used. The pH was adjusted to 6.5 by adding NaOH. The steps were performed in sequence, and one among 1 mL of buffer, 3.5 mL of the buffered monomer solution, and 0.5 mL of the buffered tyrosinase solution was added to a 15 mL tube to reach a final volume of 5 mL, followed by incubation in the dark for one week. After mixing the solution, 1 mL aliquot was retrieved and centrifuged at 11,200 ×g for 10 min in a refrigerated centrifuge (Thermo Fisher, India) at 4 °C. The FT-IR spectra were obtained by scanning the liquid samples in ATR- FTIR (FTIR–6800 JASCO, Japan). The transmittance spectra were recorded in the wavenumber range of 4000–400 cm^− 1^ with a spectral resolution of 4 cm^− 1^ and 32 scans per sample [34].

## Supplementary Information

**Additional file 1: Figure S1.** A plate depicting the brown coloration representing the oxidation of L-tyrosine by the isolate TFG5. Brown color colonies (a) indicates the isolate TFG5 oxidizes L- tyrosine and no color formation (b) indicates medium supplemented without L-tyrosine. **Figure S2.** ARDRA profiling of tyrosinase/laccase-positive isolates using AluI restriction enzyme. **Figure S3.** ARDRA profiling of tyrosinase/laccase-positive isolates using HpaII restriction enzyme. **Figure S4.** ARDRA profiling of tyrosinase/laccase-positive isolates using HaeIII restriction enzyme. **Figure S5.** A. Polymerization of p-hydroxybenzoic acid (a) control b) tyrosinase from mushroom c) tyrosinase from TFT-5. B. Polymerization of catechol (a) control b) tyrosinase from mushroom c) tyrosinase from TFT-5. C. Polymerization of ferulic acid (a) control b) tyrosinase from mushroom c) tyrosinase from TFT-5. D. Polymerization of salicylic acid (a) control b) tyrosinase from mushroom c) tyrosinase from TFT-5

## Data Availability

All data of this manuscript are included in the manuscript. The genebank accession number of laccase and tyrosinse positive isolate *Bacillus aryabhattai* TFG5 is KT956906 which has been submitted to and released by NCBI. No separate external data source is required. Any additional information required will be provided by communicating with the corresponding author via the official mail: usiva@tnau.ac.in.

## Abbreviations

ARDRA: Amplified Ribosomal DNA restriction analysis
AluI: *Arthrobacter luteus*
HpaII: *Haemophilus parainfluenza*
HaeIII: *Haemophilus aegyptius*
HCA: Hierarchical cluster analysis
TFG5: Termite fungal Garden 5
L-DOPA: L-3,4-dihydroxyphenylalanine
PLD: Pine leaves decomposed soil
WD: Wood degraded soil
FT-IR: Fourier Transformation Infrared Spectroscopy

## References

[1] Zaidi KU, Ali AS, Ali SA, Naaz I (2014). Microbial Tyrosinases: Promising Enzymes for Pharmaceutical, Food Bioprocessing, and Environmental Industry. Biochem Res Int.

[2] Sharma KK, Kuhad RC (2009). An evidence of laccases in archaea. Indian J Microbiol.

[3] Singh D, Sharma KK, Dhar MS, Virdi JS (2014). Molecular modeling and docking of novel laccase from multiple serotype of *Yersinia enterocolitica* suggests differential and multiple substrate binding. Biochem Biophys Res Commun.

[4] Parmar N, Singh KH, Sharma D, Singh L, Kumar P, Nanjundan J, Khan YJ, Chauhan DK, Thakur AK (2017). Genetic engineering strategies for biotic and abiotic stress tolerance and quality enhancement in horticultural crops: a comprehensive review. 3 Biotech.

[5] Chauhan PS, Goradia B, Saxena A. Bacterial laccase: recent update on production, properties and industrial applications. 3 Biotech. 2017;7:323.10.1007/s13205-017-0955-7. 10.1007/s13205-017-0955-7 PMC5602783 28955620

[6] Sharma KK, Kuhad RC (2008). Laccase: enzyme revisited and function redefined. Indian J Microbiol.

[7] Pretzler M, Bijelic A, Rompel A (2017). Heterologous expression and characterization of functional mushroom tyrosinase (AbPPO4). Sci Rep.

[8] Claus H, Decker H. Bacterial tyrosinases. Syst Appl Microbiol. 2006;29:–14. 10.1016/j.syapm.2005.07.012 16423650

[9] Masran R, Zanirun Z, Bahrin EK, Ibrahim MF, Lai Yee P, Abd-Aziz S (2016). Harnessing the potential of ligninolytic enzymes for lignocellulosic biomass pretreatment. Appl Microbiol Biotechnol..

[10] Guo J, Rao Z, Yang T, Man Z, Xu M, Zhang X, Yang ST (2015). Enhancement of the thermostability of *Streptomyces kathirae* SC-1 tyrosinase by rational design and empirical mutation. Enzyme Microb Technol.

[11] Pinero S, Rivera J, Romero D, Cevallos MA, Martinez A, Bolivar F, Gosset G (2007). Tyrosinase from *Rhizobium etli* is involved in nodulation efficiency and symbiosis-associated stress resistance. J Mol Microbiol Biotechnol..

[12] Kong KH, Hong MP, Choi SS, Kim YT, Cho SH (2000). Purification and characterization of a highly stable tyrosinase from *Thermomicrobium roseum*. Biotechnol Appl Biochem.

[13] Fairhead M, Thöny-Meyer L. Bacterial tyrosinases: old enzymes with new relevance to biotechnology. New Biotechnol. 2012;29:183–91. 10.1016/j.nbt.2011.05.007 21664502

[14] Hosseini-Abari A, Kim BG, Lee SH, Emtiazi G, Kim W, Kim JH (2016). Surface display of bacterial tyrosinase on spores of *Bacillus subtilis* using CotE as an anchor protein. J Basic Microbiol..

[15] Kanteev M, Goldfeder M, Chojnacki M, Adir N, Fishman A (2013). The mechanism of copper uptake by tyrosinase from *Bacillus megaterium*. J Biol Inorg Chem.

[16] Wang F, Xu Z, Wang C, Guo Z, Yuan Z, Kang H, Li J, Lu F, Liu Y (2021). Biochemical characterization of a tyrosinase from *Bacillus aryabhattai* and its application. Int J Biol Macromol.

[17] Binner E, Smidt E, Tintner J, Böhm K, Lechner P (2011). How to enhance humification during composting of separately collected biowaste: impact of feedstock and processing. Waste Manage Res.

[18] Zavarzina A, Lisov A, Zavarzin A, Leontievsky A. Fungal oxidoreductases and humification in forest soils. In: Soil Enzymology. Berlin: Springer; 2010. p. 207–28.

[19] Zavarzina AG, Lisov AA, Zavarzin AA, Leontievsky AA: Fungal Oxidoreductases and Humification in Forest Soils. In: Soil Enzymology. Edited by Shukla G, Varma A, Berlin, Heidelberg: Springer Berlin Heidelberg; 2011: 207-28. 10.1007/978-3-642-14225-3$411.

[20] Muniraj I, Shameer S, Ramachandran P, Uthandi S: *Bacillus aryabhattai* TFG5-mediated synthesis of humic substances from coir pith wastes. In: Microbial cell factories. 2021:20;48. 10.1186/s12934-021-01538-x PMC7891170 33596930

[21] Ba S, Vinoth Kumar V. Recent developments in the use of tyrosinase and laccase in environmental applications. Crit Rev Biotechnol. 2017;1–14. 10.1080/07388551.2016.1261081 10.1080/07388551.2016.1261081 28330374

[22] Janusz G, Kucharzyk KH, Pawlik A, Staszczak M, Paszczynski AJ (2013). Fungal laccase, manganese peroxidase and lignin peroxidase: gene expression and regulation. Enzyme Microb Technol.

[23] Sklarz MY, Angel R, Gillor O, Soares MIM (2009). Evaluating amplified rDNA restriction analysis assay for identification of bacterial communities. Antonie van Leeuwenhoek.

[24] Ayeni FA, Adeniyi BA, Ogunbanwo ST, Tabasco R, Paarup T, Pelaez C, Requena T (2009). Inhibition of uropathogens by lactic acid bacteria isolated from dairy foods and cow’s intestine in western Nigeria. Arch Microbiol..

[25] Dalfard AB, Khajeh K, Soudi MR, Naderi-Manesh H, Ranjbar B, Sajedi RH (2006). Isolation and biochemical characterization of laccase and tyrosinase activities in a novel melanogenic soil bacterium. Enzyme Microb Technol.

[26] Desentis-Mendoza RM, Hernandez-Sanchez H, Moreno A, del c Rojas E, Chel-Guerrero L, Tamariz J, Jaramillo-Flores ME (2006). Enzymatic polymerization of phenolic compounds using laccase and tyrosinase from *Ustilago maydis*. Biomacromolecules.

[27] Gerke J (2018). Concepts and Misconceptions of Humic Substances as the Stable Part of Soil Organic Matter: a Review. Agronomy.

[28] Battaini G, Monzani E, Casella L, Lonardi E, Tepper AW, Canters GW, Bubacco L (2002). Tyrosinase-catalyzed oxidation of fluorophenols. J Biol Chem..

[29] Winder AJ (1994). A stopped spectrophotometric assay for the dopa oxidase activity of tyrosinase. J Biochem Biophys Methods.

[30] Leonowicz A, Grzywnowicz K (1981). Quantitative estimation of laccase forms in some white-rot fungi using syringaldazine as a substrate. Enzyme Microb Technol.

[31] Bradford MM (1976). A rapid and sensitive method for the quantitation of microgram quantities of protein utilizing the principle of protein-dye binding. Anal Biochem.

[32] Stakenborg T, Vicca J, Butaye P, Maes D, De Baere T, Verhelst R, Peeters J, de Kruif A, Haesebrouck F, Vaneechoutte M (2005). Evaluation of amplified rDNA restriction analysis (ARDRA) for the identification of Mycoplasma species. BMC Infect Dis.

[33] Kobayashi S, Higashimura H (2003). Oxidative polymerization of phenols revisited. Prog Polymer Sci.

[34] Fooken U, Liebezeit G (2003). An IR study of humic acids isolated from sediments and soils. Senckenbergiana Maritima.

