# Supplementary material for: Tyrosinase and laccase-producing Bacillus aryabhattai TFG5 and its role in the polymerization of phenols
Source: BMC Microbiol. 2021 Jun 22;21:187. doi: 10.1186/s12866-021-02258-3 (PMC8220707; doi:10.1186/s12866-021-02258-3)
Supplement: Supplementary file 1 — Additional file 1: Figure S1. A plate depicting the brown coloration representing the oxidation of L-tyrosine by the isolate TFG5. Brown color colonies (a) indicates the isolate TFG5 oxidizes L- tyrosine and no color formation (b) indicates medium supplemented without L-tyrosine. Figure S2. ARDRA profiling of tyrosinase/laccase-positive isolates using AluI restriction enzyme. Figure S3. ARDRA profiling of tyrosinase/laccase-positive isolates using HpaII restriction enzyme. Figure S4. ARDRA profiling of tyrosinase/laccase-positive isolates using HaeIII restriction enzyme. Figure S5. A. Polymerization of p-hydroxybenzoic acid (a) control b) tyrosinase from mushroom c) tyrosinase from TFT-5. B. Polymerization of catechol (a) control b) tyrosinase from mushroom c) tyrosinase from TFT-5. C. Polymerization of ferulic acid (a) control b) tyrosinase from mushroom c) tyrosinase from TFT-5. D. Polymerization of salicylic acid (a) control b) tyrosinase from mushroom c) tyrosinase from TFT-5 [file 12866_2021_2258_MOESM1_ESM.doc]

**Tyrosinase and laccase-producing *Bacillus aryabhattai* TFG5 and its role in the polymerization of phenols**

Iniyakumar Muniraj^1^, Syed Shameer^1^, Sivakumar Uthandi^1^

Biocatalysts lab., Department of Agricultural Microbiology, Tamil Nadu Agricultural University, Coimbatore 641 003

**
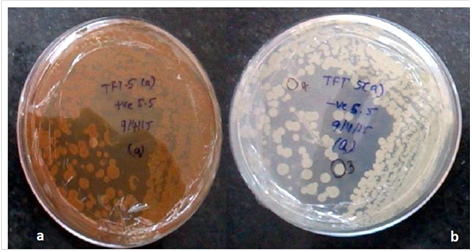
**

Figure S1. A plate depicting the brown coloration representing the oxidation of L-tyrosine by the isolate TFG5. Brown color colonies (a) indicates the isolate TFG5 oxidizes L- tyrosine and no color formation (b) indicates medium supplemented without L-tyrosine.

Figure S2**. ARDRA profiling of tyrosinase/laccase-positive isolates using AluI restriction enzyme.**

**Figure S3. ARDRA profiling of tyrosinase/laccase-positive isolates using HpaII restriction enzyme.**

**
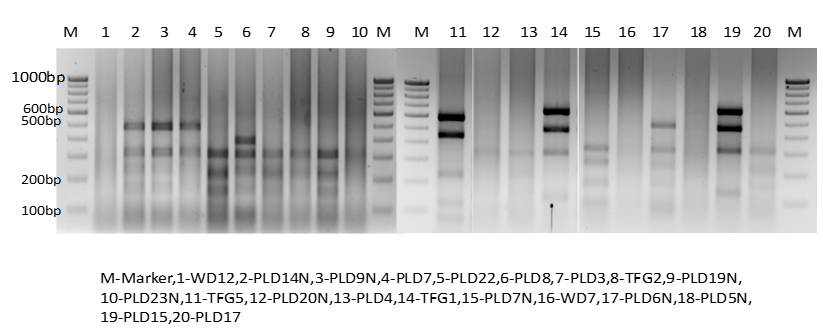
**

**Figure S4. ARDRA profiling of tyrosinase/laccase-positive isolates using HaeIII restriction enzyme.**


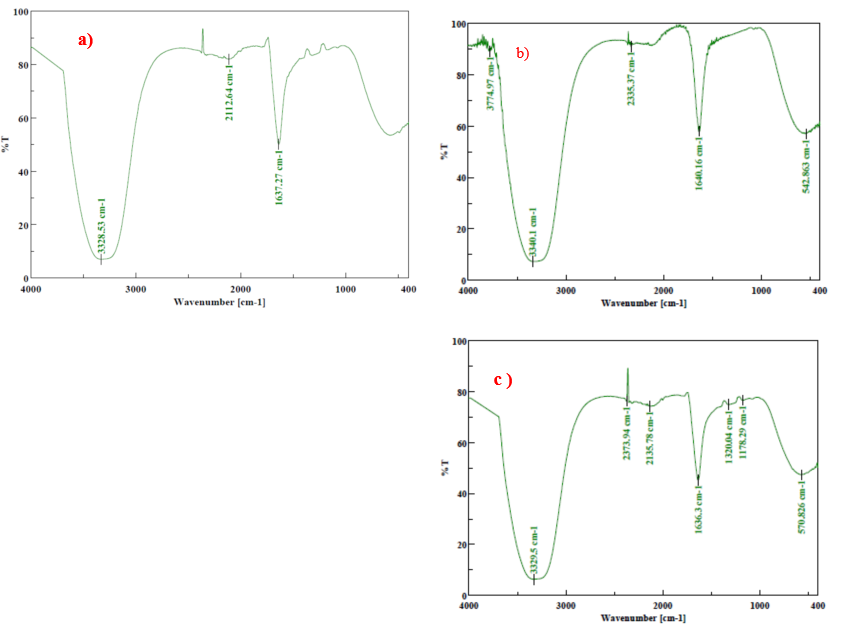


Fig.S5 A. Polymerization of p-hydroxybenzoic acid (a) control b) tyrosinase from mushroom c) tyrosinase from TFT-5


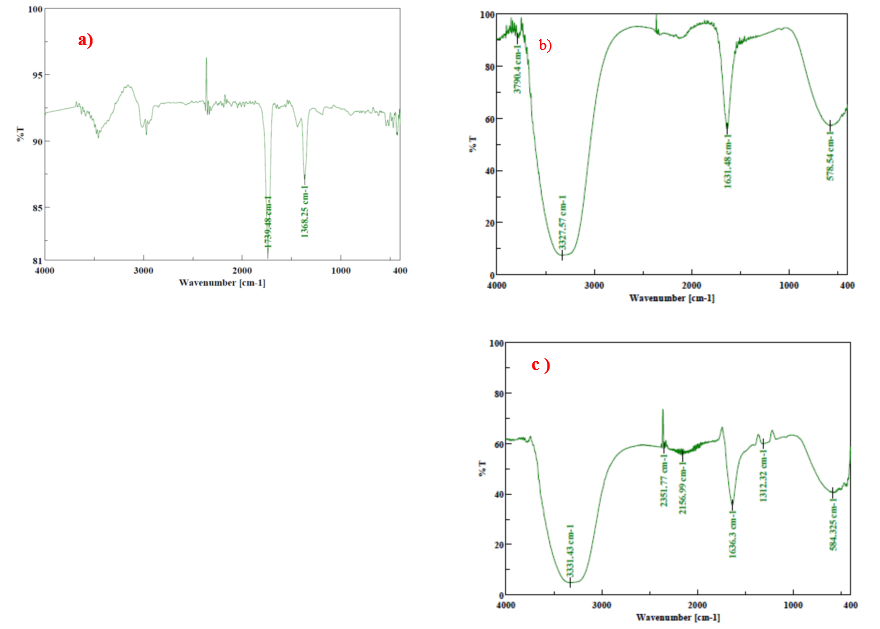


Fig.S5 B. Polymerization of catechol (a) control b) tyrosinase from mushroom c) tyrosinase from TFT-5

**
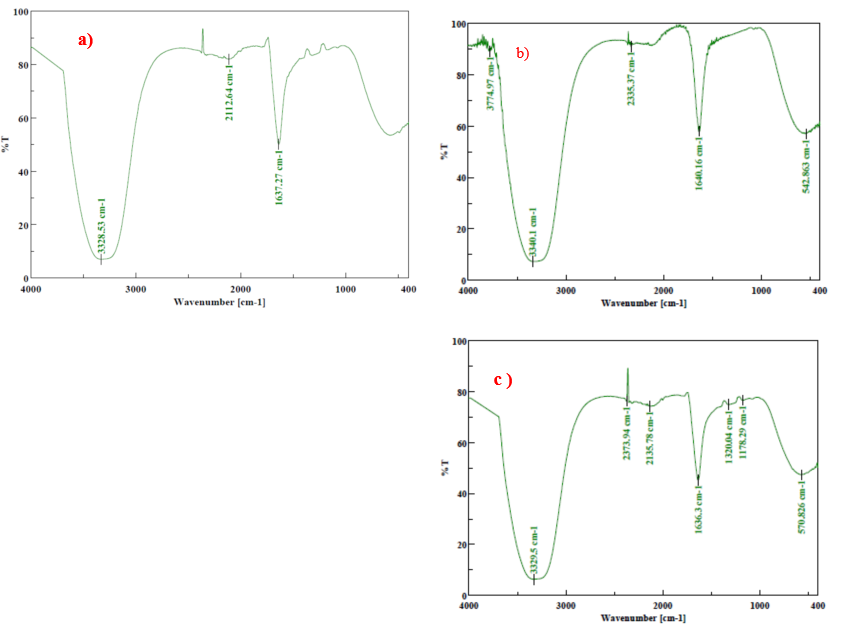
**

Fig.S5 C. Polymerization of ferulic acid (a) control b) tyrosinase from mushroom c) tyrosinase from TFT-5

**
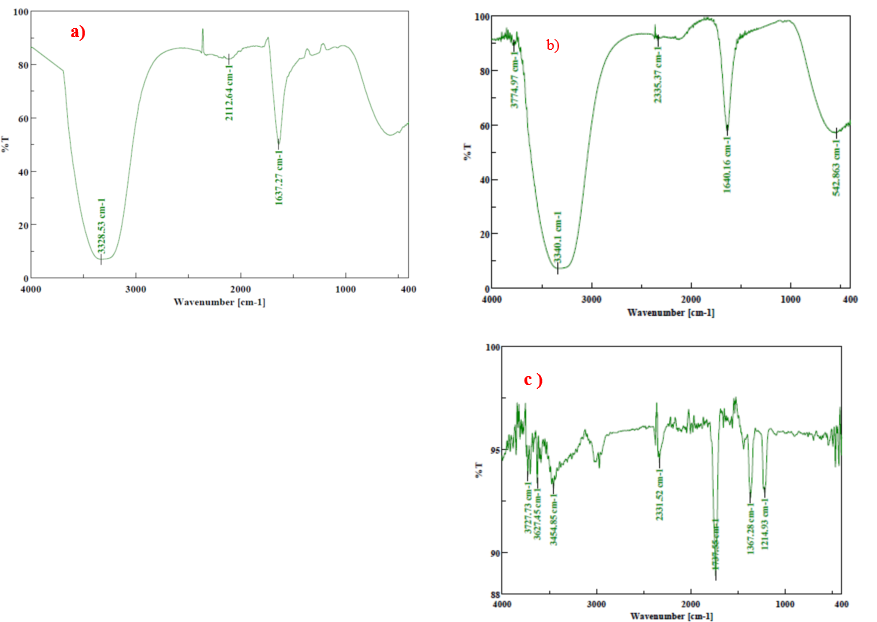
**

Fig.S5 D. Polymerization of salicylic acid (a) control b) tyrosinase from mushroom c) tyrosinase from TFT-5
